# Super-resolved visualization of single DNA-based tension sensors in cell adhesion

**DOI:** 10.1038/s41467-021-22606-1

**Published:** 2021-05-04

**Authors:** Thomas Schlichthaerle, Caroline Lindner, Ralf Jungmann

**Affiliations:** 1 Faculty of Physics and Center for Nanoscience, Ludwig Maximilian University, Munich, Germany; 2 Max Planck Institute of Biochemistry, Martinsried, Germany

**Keywords:** Fluorescence imaging, Nanostructures, Single-molecule biophysics

## Abstract

Cell-extracellular matrix sensing plays a crucial role in cellular behavior and leads to the formation of a macromolecular protein complex called the focal adhesion. Despite their importance in cellular decision making, relatively little is known about cell-matrix interactions and the intracellular transduction of an initial ligand-receptor binding event on the single-molecule level. Here, we combine cRGD-ligand-decorated DNA tension sensors with DNA-PAINT super-resolution microscopy to study the mechanical engagement of single integrin receptors and the downstream influence on actin bundling. We uncover that integrin receptor clustering is governed by a non-random organization with complexes spaced at 20–30 nm distances. The DNA-based tension sensor and analysis framework provide powerful tools to study a multitude of receptor-ligand interactions where forces are involved in ligand-receptor binding.

## Abstract (web summary)

Relatively little is known about cell-matrix interactions and the intracellular transduction of an initial ligand-receptor binding event on the single-molecule level. Here authors combine ligand-decorated DNA tension sensors with DNA-PAINT super-resolution microscopy to study the mechanical engagement of single integrin receptors and the downstream influence on actin bundling.

## Introduction

Cell-extracellular matrix sensing plays an important role in cellular behavior, immune homeostasis and development^1,2^. Sensing is mainly mediated by integrin receptors^3^ upon engagement of extracellular ligands such as fibronectin or collagen, which leads to local integrin clustering^4^. The interaction with matrix ligands subsequently leads to the recruitment of a macromolecular protein complex called the focal adhesion, which consists of hundreds of proteins^5–7^. The focal adhesion complex is organized in different horizontal layers, ultimately coupling the actin cytoskeleton to the extracellular environment^8^. Two proteins in particular, talin and kindlin, are of major importance for the cellular attachment process^9^, with talin providing a direct force-transduction link between the beta-integrin tail and the actin network as shown in an earlier study using genetically-encoded protein-based force sensors^10^. A multitude of tools has been developed to probe tension during cell attachment and to study the interaction of cells with their extracellular environment via externally templated probes. Traction force microscopy, for example, uses the displacement of beads embedded in a gel surrounding the cell and thus allows to track mechanical forces exerted on the extracellular matrix^11–14^. The displacement of the beads can furthermore be measured via super-resolution microscopy^15–17^, which allows to increase the gel-embedded particle density for considerably improved higher-resolution mapping of forces. However, while increasing spatial resolution, this approach still integrates forces over several tens of nanometers and falls short of the ultimate goal to interrogate and resolve forces between true single ligand-receptor pairs. To address this issue, extracellular protein-based Förster resonance energy transfer (FRET) sensors were developed to measure the mechanical tension of integrins interacting with their extracellular matrix ligands^18–20^. This advance enabled the quantification of mechanical forces on the single-molecule level and map subpopulations bearing different loads within adhesion structures^21,22^. Complementary approaches to analyze the engagement of the cell with their extracellular environment used DNA-based probes functionalized with cRGD motifs^23–25^. DNA-based sensors, compared to protein sensors, allow for more modular and flexible tuning of the force regime^26^. Notably, a recent study by Brockman et al. used DNA-PAINT super-resolution imaging to super-resolve cellular traction forces in living cells^27^. This approach is very promising and provides insights at thus far unprecedented spatiotemporal levels. However, live-cell imaging to date does not allow researchers to observe ligand-receptor engagement at the true single-protein level. While the field has seen tremendous advances through technical improvements mentioned above, future studies would benefit from techniques that achieve even higher spatial resolutions to improve the characterization of mechanically engaged ligand-receptor pairs and further probe their spatial organization at thus fare elusive length scales.

**Fig. 1:**
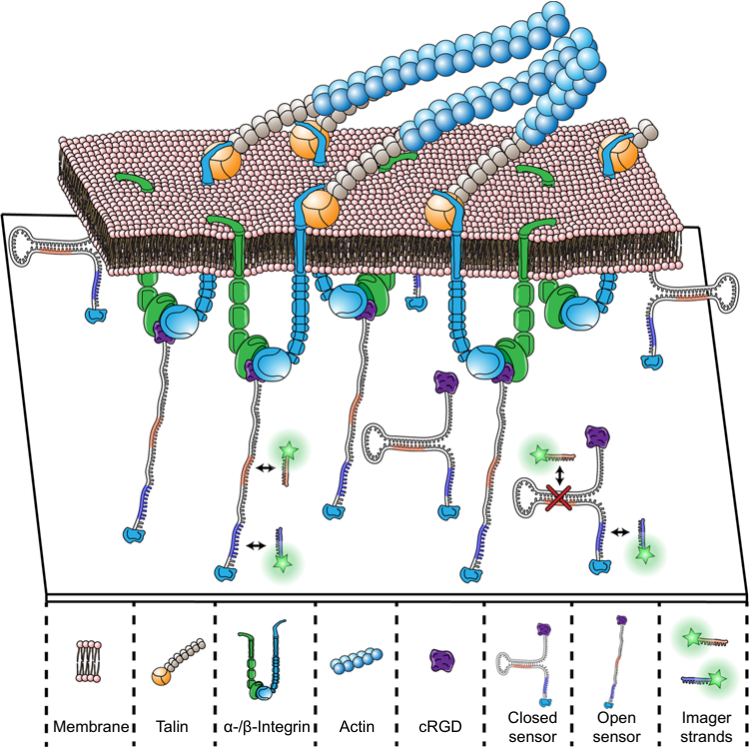
Single-receptor tension sensor. Biotinylated DNA-based hairpin sensors are immobilized on a streptavidin-coated glass surface. The location of the sensor is super-resolved via DNA-PAINT microscopy by sequence-specific targeting of the blue-colored DNA sequence. Upon cell attachment and integrin-binding of the cRGD motif on the sensor, the hairpin is opened, and the extended sensor conformation can be detected by DNA-PAINT through targeting of a previously sequestered orange-colored sequence. Subsequent binding of focal adhesion proteins such as talin to the beta-integrin tail eventually leads to a full force transduction path and coupling to intracellular actin filaments.

We here developed a DNA-based molecular tension sensor, which carries a sequestered DNA binding site for DNA-PAINT^28–30^ super-resolution microscopy, which is revealed upon mechanical unfolding as a result of binding of the cyclic arginine-glycine-aspartatic (cRGD) motif to an integrin receptor (Fig. 1). DNA-PAINT uses the transient binding of dye-labeled oligonucleotides (called imager strands) to their complementary target sites (called docking strands) to create an apparent target blinking typically harnessed in single-molecule localization microscopy. Using the DNA-based sensor, we employ the molecular-scale resolution of DNA-PAINT to quantify the absolute position, pattern, and density of true single ligands on a glass surface and unveil that mechanically unfolded sensors are not randomly distributed in focal adhesion areas but are in fact molecularly clustered with characteristic distances of about 20–30 nm. Finally, we show how this receptor clustering translates to the force-generating intracellular cytoskeletal architecture by multiplexed visualization of both the mechanically strained force sensors and the cellular actin network.

## Results

### DNA-based tension sensor for super-resolution microscopy

We devised a DNA-based tension sensor (Fig. 1), consisting of a Biotin modification for surface attachment, a single-stranded sequence stretch featuring a DNA-PAINT docking site (blue-colored sequence in Fig. 1) for sensor localization, a hairpin-sequestered orthogonal docking site (orange-colored sequence in Fig. 1) to visualize mechanically unfolded sensors, and finally a cRGD peptide modification for binding to the integrin receptors. For the design of the hairpin stem, we took several aspects into consideration. The stem should open as soon as a specific force threshold was reached and reveal a previously sequestered docking site designed for fast and efficient DNA-PAINT super-resolution imaging^31,32^. Based on our stem design, we estimated the unzipping force to be ~9 pN (see methods for details), which is well below the reported force threshold for initial integrin adhesion^33^, estimated to be ~40 pN. To approximate if the sensor is closed at seeding (37 °C) and imaging conditions (20 °C), we performed a NUPACK^34^ secondary structure prediction analysis (Supplementary Fig. 1), which yielded a closed hairpin for both conditions. For the synthesis of the DNA-based hairpin sensor, we first conjugated a biotinylated and DBCO-modified DNA oligonucleotide with a commercially available Azide-labeled cRGD motif and purified it via anion-exchange chromatography. The cRGD-labeled strand was then immobilized on a biotinylated PEG surface (see methods for details). Achieving sufficiently high surface ligand density has been shown to be crucial for cellular attachment in earlier studies^35–37^.

### Single-molecule surface density and pattern

**Fig. 2:**
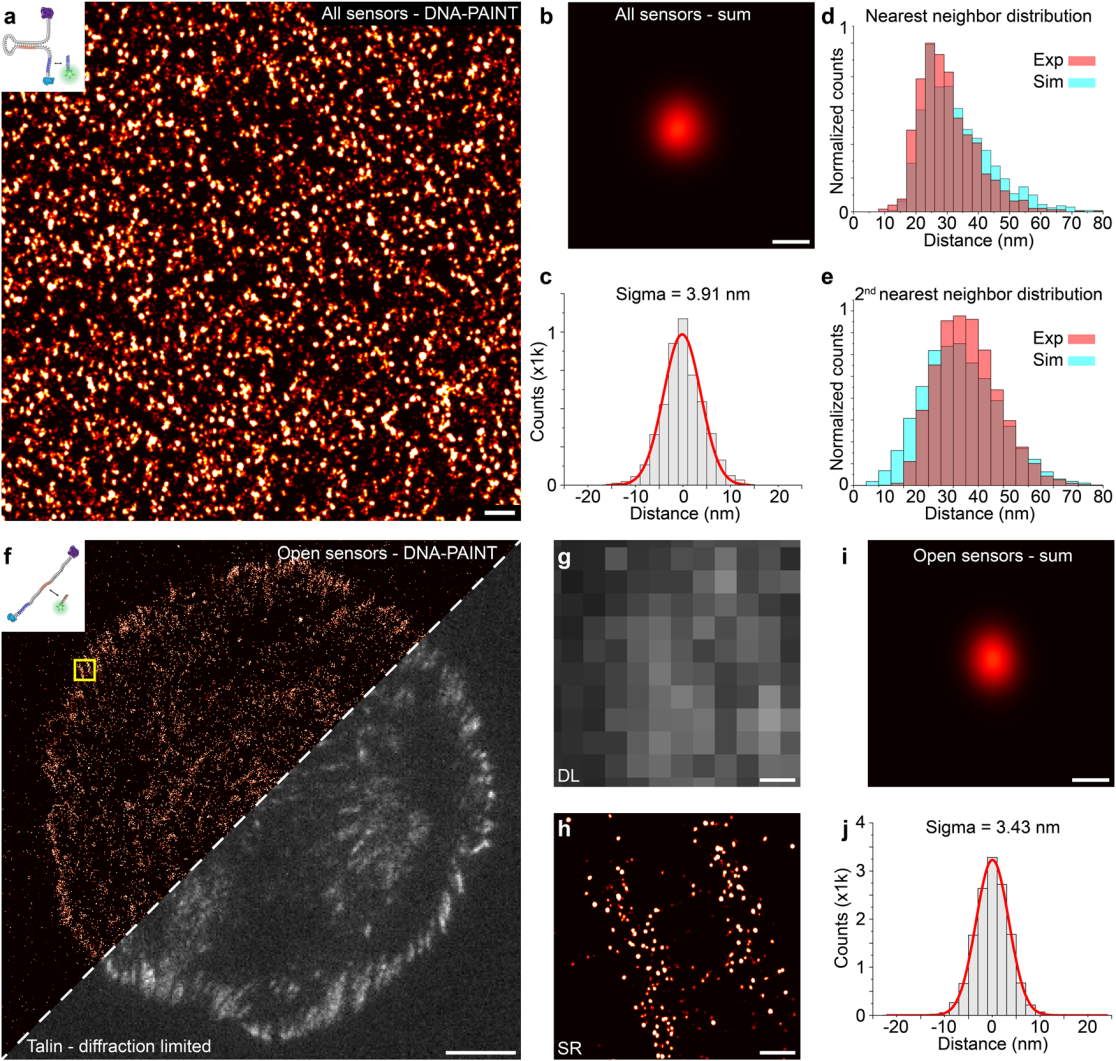
DNA-PAINT imaging of surface-immobilized and mechanically unfolded sensors. **a** Super-resolved overview image of surface-immobilized, closed hairpin sensors (targeting of blue docking sequence). **b** Center-of-mass-aligned sum image of 100 single sensor signals. **c** Cross-sectional histogram yields localization precisions of 3.91 nm. **d** Experimentally determined Nearest Neighbor Distance (NND) distribution of the single sensor sites (red) and simulated NND distribution assuming Complete Spatial Randomness (CSR) at the same surface density of 422 molecules per µm^2^ (cyan) confirms random distribution of DNA hairpin sensors on the surface. **e** Experimental 2^nd^ nearest neighbor distance distribution (red) and corresponding random distribution with the same molecular density (cyan). **f** DNA-PAINT super-resolved image of extended DNA hairpin sensors (targeting the orange sequence) 25 min after seeding of fibroblasts on the hairpin-functionalized cover slip (left). Corresponding diffraction-limited talin signal (right) highlights localization of extended hairpin sensors to focal adhesion sites. **g** Zoom-in of highlighted area in **f** of the diffraction-limited talin signal. **h** Corresponding zoom-in of the super-resolved extended hairpin sensors. **i** Center-of-mass-aligned sum image of 100 single extended sensor signals. **j** Cross-sectional histogram yields localization precisions of 3.43 nm, highlighting that indeed single hairpins (and thus engaged cRGD ligands) are visualized. Scale bars: 100 nm (**a**), 10 nm (**b, i**), 5 µm (**f**), 200 nm (**g**, **h**).

To evaluate surface density and nanoscale ligand pattern, we imaged the permanently accessible part (blue sequence) of our DNA hairpin sensor with DNA-PAINT (Fig. 2a) and analyzed the achievable resolution and nearest neighbor distances (Fig. 2b–e). To estimate the localization precision of single ligands, 100 single sensors were selected and aligned by their center of mass to create a sum image (Fig. 2b). Subsequent cross-sectional histogram analysis yielded an overall localization precision of 3.91 nm, in good agreement with the average localization precision of 4.1 nm obtained through NeNA analysis^38^ (Fig. 2c and Supplementary Fig. 2). qPAINT analysis shows a unimodal distribution for the number of binding events per site^39^, supporting the claim that we indeed are able to visualize single ligands on the surface (Supplementary Fig. 3). For further quantitative pattern analysis of single-ligand positions, we applied a modified Ripley’s K function using a gradient ascent to find the center positions of the sensors from localization clouds and performed subsequent filtering of the detected sites to remove unspecific signals (see methods and Supplementary Fig. 4). We found a molecular density of 422 ligands per µm^2^, which translates to a mean nearest neighbor distance (NND) between individual ligands of 30 nm ± 9 nm. To test for Complete Spatial Randomness (CSR) of the ligand positions, we compared our experimental NND distribution with a CSR simulation performed with the same molecular density and obtained a similar distribution and mean NND of 33 nm ± 11 nm (*p* = 0.71 n.s., two-sided *t*-test) (Fig. 2d and Supplementary Fig. 5). To further rule out any higher order assemblies, we compared the 2^nd^ nearest neighbor distribution of our experimental data (46 nm ± 13 nm) with a CSR simulation (52 nm ± 15 nm) and again observed no differences (*p* = 0.80, n.s., two-sided *t*-test) (Fig. 2e).

### Imaging of mechanically unfolded sensors

After this initial characterization of our sensors on the surface, we next seeded Talin-deficient fibroblast cells, which were reconstituted with YPET-tagged Talin-1 (see methods for details) for 25 min on a DNA hairpin-functionalized surface, allowing the cells to form focal adhesions. In control experiments on passivated, non-functionalized surfaces (Supplementary Fig. 6), the cells did not form adhesions. After 25 min of cell attachment, we performed DNA-PAINT imaging of fixed and permeabilized cells, targeting the permanently accessible and the sequestered binding site (Supplementary Fig. 7) and observed a clear correlation of extended hairpin sensor signals with the diffraction-limited signal of the adhesion marker Talin-1 (Fig. 2f–h). We note that we do observe some open hairpin sensors in the apparent absence of cell adhesion, however we only detected 7 ± 7 ligands per µm^2^, leading to a negligible “unspecific” background signal of approximately 2%. To estimate the localization precision of single extended sensors and probe if we are indeed observing single engaged ligands (similar to the closed sensor case from above), 100 single extended sensors were selected and aligned by their center of mass to create a sum image (Fig. 2i). Subsequent cross-sectional histogram analysis yielded an overall localization precision of 3.43 nm, in good agreement with the average localization precision of 4.0 nm obtained through NeNA analysis^38^ (Fig. 2j and Supplementary Fig. 8), supporting our ability to visualize single mechanically unfolded tension sensors.

**Fig. 3:**
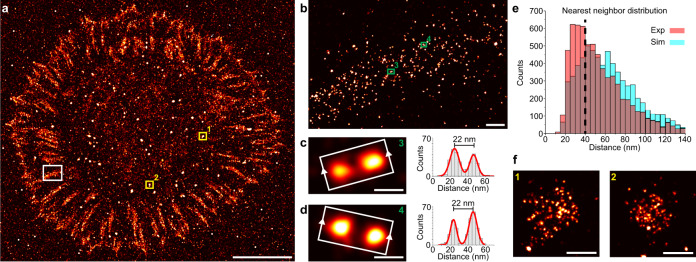
Nanoscale pattern analysis of open hairpin sensors. **a** Super-resolved DNA-PAINT image of open hairpin sensors as proxy for mechanically engaged ligand-receptor pairs, enriched in focal adhesions. **b** Zoom-in of the highlighted area (white) in **a**. **c**, **d** Zoom-ins of the highlighted areas (green) from **b** reveal closely spaced sensors (left), approximately 22 nm apart (right). **e** Comparison of experimental NND distribution of extended hairpins within focal adhesion areas selected by colocalization with diffraction-limited talin signal and NND of random simulated sites with same molecular density of 88.5 molecules per µm^2^ reveals molecular clustering below 40 nm of extended sensors. **f** Zoom-in of two nanoclusters highlighted in yellow in **a** in non-adhesion areas were found to have an average diameter of 228.3 nm. Scale bars: 10 µm (**a**), 300 nm (**b**), 20 nm (**c**, **d**), 200 nm (**f**).

As integrin clustering plays an important role in focal adhesion formation, we next evaluated the molecular density and localization pattern of open sensors in focal adhesions (Fig. 3 and Supplementary Fig. 9). We found a molecular density of 89 ± 34 per µm^2^ (*n* = 4) of unfolded sensors within the adhesion area with single sensors spaced as close as 22 nm (Fig. 3b–d). The sensitivity of the DNA hairpin to open under a specific force upon ligand-receptor binding and mechanical tension allows us to determine the aggregated minimal force within an adhesion area. The molecular density we obtained translates to minimal aggregated forces of at least 800 pN per µm^2^ employed by the cell on the extracellular environment (see methods) within adhesion areas, which is in good agreement with previous reports^40^. We note, however that our current sensor design only detects mechanically engaged integrins that exert forces in excess of 9 pN, without being able to precisely state the actual force over the mechanical linkage between individual receptor-ligand pairs. Additionally, previous work suggests that integrin-ligand interactions can bear lower forces^18,21^. It was furthermore previously shown that fixation can lead to imaging artefacts^41^. Considering all these factors, we note that our hairpin sensor is only able to determine the minimal aggregated force as stated above.

### Mechanically unfolded sensors are distributed non-randomly

Next, we further investigated if the pattern of unfolded DNA hairpin sensors at focal adhesions is characterized by Complete Spatial Randomness or potentially mediated by an underlying non-random molecular clustering process^42^ at characteristic distances. To answer this, we compared our experimental NND distribution for open hairpin sensors with a CSR simulation performed with the same molecular density (Fig. 3e). We observed a clear deviation of the experimental NND distribution from the CSR simulated case for distances closer than 40 nm, suggesting that in fact a molecular spatial association process leads to receptor clustering at these length scales. Previous work using nanotemplated cRGD ligands on gold arrays showed that ligand spacing below 60–70 nm plays a crucial role and is essential for cellular attachment^35,37^. Our data supports this claim by further visualizing a non-random organization of mechanically engaged ligand-receptor pairs. By quantitatively analyzing the fraction of open sensors with an NND closer than 40 nm, we obtained a 34% higher fraction in the experimental data compared to the CSR simulation, with a peak at approximately 30 nm (*p* = 0.04*, two-sided *t*-test). Earlier work using super-resolution techniques showed that active and inactive integrin populations segregate into distinct nanoclusters within adhesion areas, however our sensors only detect a subfraction^43^ (namely the one that exerts more than 9 pN on their ligand) of activated receptors. Within this fraction, we do observe non-random organization. We furthermore visualized dense and distinct nanoclusters of mechanically engaged receptors with an average diameter of 228 nm ± 60 nm within the cell area (Fig. 3f). These clusters were observed in multiple cells and were heterogenous in shape, however they did show specific signal of open hairpins (Supplementary Figs. 10–12).

### Correlation to the actin network

**Fig. 4:**
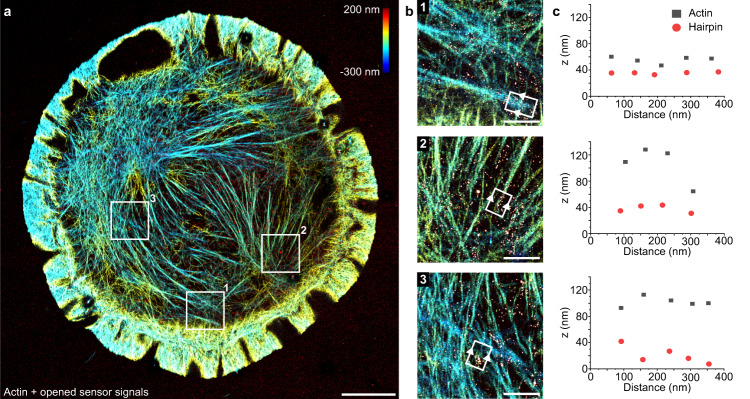
Two-target 3D super-resolution characterization of hairpin sensors and actin cytoskeleton. **a** Super-resolved actin network using Lifeact-Cy3B (color-coded according to z-position) overlayed with extended sensor signal (red). **b** Zoom-ins of highlighted areas in **a**. **c** Z-separation between extended hairpin sensor positions and the actin filaments indicates that the filaments can be found above the hairpin at various distances. Top: Extended sensor is approximately 20 nm apart from the filaments. Middle: Mean filament position is approximately 80 nm above the extended sensor. Bottom: Mean filament position is again 60–80 nm above the extended sensor. Scale bars: 5 µm (**a**), 1 µm (**b**).

Finally, to further investigate the transduction of extracellular ligand binding and mechanical tension to the intracellular nanoscale protein architecture, we correlated the actin cytoskeleton with the signal of engaged ligands in a 3D DNA-PAINT experiment (Fig. 4 and Supplementary Figs. 13 and 14). We performed two rounds of Exchange-PAINT, visualizing extended DNA hairpin probes with classical DNA-PAINT and the actin cytoskeleton using a Cy3B-modified version of Lifeact^44^. This allowed us (post-channel alignment) to visualize absolute axial positions of extended DNA hairpins in correlation with the underlying actin cytoskeleton network without chromatic aberrations and with high z-resolution (Fig. 4a). We measured the mean position in the axial and vertical direction of all occurring localizations in a 75 nm window (see methods for details) and were able to assign the signal from extended DNA sensor clusters to individual actin bundles and measure the respective height distribution of sensor and actin filaments along the axial direction (Fig. 4b, c). Actin filaments were found 20–80 nm above the open hairpin signal, in good agreement with earlier studies^8^ reporting a layered architecture of focal adhesion molecules. Additionally, we found colocalization of open hairpin clusters throughout the cell membrane with clusters of actin (Supplementary Fig. 15).

## Discussion

We have developed a DNA-based molecular tension sensor, which carries a sequestered DNA binding site for DNA-PAINT super-resolution microscopy that is revealed upon mechanical engagement of a cRGD motif through binding to an integrin receptor. In comparison to a recent study by Brockman et al., which used DNA-PAINT to super-resolve molecular tension in live cells^27^, we here focused on the visualization and analysis of patterns of open sensors at a fixed time point with true single-ligand resolution. The molecular-scale spatial resolution of DNA-PAINT enables the quantification of absolute position, pattern, density, and tension state of these single-molecule sensors. Quantification and comparison of the observed pattern with simulations revealed that closed sensors (and thus ligands) are randomly distributed prior to cell attachment. After cell adhesion and mechanical unfolding of cRGD ligands by integrins, we found that single unfolded ligand sites are overall correlated with areas of focal adhesion as demonstrated with the colocalization with Talin as an adhesion marker. By resolving single receptor-ligand sites, which are actively unfolded and take part in the force transduction pathway, we observed unfolded sensors spaced as close as 20 nm apart. Subsequent quantitative analysis of their nearest neighbor distance distribution revealed that integrin receptor clustering does not seem to follow a mere random distribution but must be mediated by an underlying molecular clustering process. The unique combination of DNA-based force sensors with DNA-PAINT readout offers direct evidence for nanoscale clustering between individual ligand-receptor pairs during adhesion formation. Finally, we show how this receptor clustering translates to the force-generating intracellular cytoskeleton by multiplexed visualization of force sensors together with the actin network. In the future, super-resolved imaging of our tension sensor could be combined with the multiplexed visualization of other adhesion markers such as paxillin or vinculin, as was previously shown with protein-based sensors^45^, to map individual adhesion units with single-protein resolution.

In conclusion, we could show that it is possible to resolve single ligands that are actively mechanically unfolded by the cellular machinery. While previous works nanotemplated ligands in various patterns and analyzed cellular behavior^46^, we can now visualize, which ligands actively interact with their respective receptors and downstream signaling cascade components. Looking ahead, we envision that our system could be combined with specific nanotemplated ligand islands (realized with e.g. DNA origami nanostructures^46–48^) to understand, how precise local arrangement might modulate signaling outcome. Our sensors not only open the possibility to study single receptor-ligand binding under mechanical tension and their influence on pattern formation but could also shed light on the co-recruitment of different factors upon single-ligand binding and could thus find diverse applications for different receptor-ligand pairs and their influence on intracellular signaling.

## Methods

### Hairpin design and conjugation

DBCO-modified hairpin DNA sensors were ordered from Biomers.net and reacted at 10–20 nM concentration with 10x excess of Azide-cRGD (Peptides International, cat. no. RGD-3759-PI). Conjugation was carried out overnight at 4 °C in 1 × PBS in a total volume of 100 µl and subjected to Anion-Exchange Chromatography (GE Healthcare, Resource Q column) using a gradient from 1 × PBS to 1 × PBS + 1 M NaCl over the course of 25 min. Peak fractions were collected and dialyzed four times (2 × 1 h, 1× overnight, 1 × 1 h) using a 1.8 l Millipore water reservoir via Slide-A-Lyzer Mini Dialysis Devices with a molecular weight cutoff of 3.5 kDa (Thermo Fisher Scientific, cat. no. 66330). Dialyzed DNA-cRGD was concentrated via vacuum centrifugation to a final sample volume of 100 µl. Successfully conjugated DNA hairpin sensors were stored at −20 °C until further use in cell attachment experiments.

### Surface preparation

PEG surfaces were prepared as previously reported^49^. In short, the microscopy coverslips (no. 1.5 high precision, 24 × 60 mm^2^, Marienfeld, cat. no. 0107032) were placed into a Teflon-based custom-made slide holder, rinsed twice and bath-sonicated in Milli-Q water for 10 min. The rinsing and washing process was repeated with methanol and acetone. For surface activation, the coverslips were bath-sonicated in 1 M KOH for 20 min and rinsed with Milli-Q water afterwards. The slides were then blow-dried with nitrogen and 170 ml of methanol was mixed with 10 ml acetic acid as well as 20 ml aminosilane (Sigma-Aldrich, cat. no. 104884-100 ML) and was immediately poured over the slide holder. The reaction was incubated for 20 min in the dark. The coverslips were then washed two times with methanol and water for 1–2 min per wash. After blow-drying with nitrogen, the aminosilanized coverslips were stored under Argon atmosphere for <2 weeks until further use. Prior to use, glass coverslips were attached to self-adhesive ibidi sticky slides in the 6-channel layout (ibidi, cat. no. 80608). NHS-mPEG750 (Rapp Polymere, cat. no. 12750-35) was mixed with NHS-Biotin (Thermo Fisher Scientific, cat. no. 20217) in 1× PBS for final concentrations of 500 nM and 5 µM at a final volume of ~150 µl and incubated for 2 h in the dark in the channels. The channels were afterwards washed with 1 ml of 1× PBS and incubated with Neutravidin (Thermo Fisher Scientific, cat. no. 31000) at a final concentration of 0.5 mg/ml for 20 min. After washing with 1 ml of 1× PBS, the channels were incubated for 45 min with 50–100 nM of cRGD DNA-Hairpin probe and subsequently washed with 1 ml of 1× PBS and 1 ml of serum-free cell medium and immediately used for cell seeding.

### Cell culture

Cells were maintained in high glucose DMEM (Thermo Fisher Scientific, cat. no. 31966047) supplemented with 1% Penicillin/Streptomycin and 10% Fetal Bovine Serum and passaged every other day. hTalin-YPet[447] was stably expressed via retroviral infection in double knockout fibroblasts deficient for talin-1 and talin-2 (Tln1-/2 Tln2-/-; dKO) as previously described^10^. Cells were seeded on pegylated cRGD-DNA surfaces at a concentration of 400,000 cells per ml and allowed to attach for 25 min.

### Immunostaining

After 25 min, cells were washed three times with 1× PBS and immediately fixed with prewarmed 4% PFA (Electron Microscopy Sciences, cat. no. 15710) in 1× PBS for 20 min. After washing with 1 ml of 1× PBS, cells were blocked and permeabilized with sterile-filtered 3% BSA (Sigma-Aldrich, cat. no. A4503-10g) and 0.25% Triton X-100 (Carl Roth, cat. no. 6683.1) for 90 min. Cells were washed again with 1 ml of 1× PBS and 90 nm gold particles (cytodiagnostics, cat. no. G-90-100) were added to the chamber in a 1:1 ratio in 1× PBS for 5 min. After washing with 1 ml of 1× PBS, cells were immediately imaged.

### Super-resolution microscopy setup

Fluorescence imaging was carried out on an inverted Nikon Eclipse Ti microscope (Nikon Instruments) with the Perfect Focus System, applying an objective-type TIRF configuration with an oil-immersion objective (Apo SR TIRF 100×, NA 1.49, Oil). Two lasers were used for excitation: 488 nm (200 mW, Toptica iBeam smart) or 561 nm (200 mW, Coherent Sapphire). The laser beam was passed through a cleanup filter (ZET488/10x or ZET561/10x, Chroma Technology) and coupled into the microscope objective using a beam splitter (ZT488rdc or ZT561rdc, Chroma Technology). Fluorescence light was spectrally filtered with two emission filters (ET525/50 m and ET500lp for 488 nm excitation and ET600/50 and ET575lp for 561 nm excitation, Chroma Technology) and imaged on a sCMOS camera (Andor Zyla 4.2) without further magnification, resulting in an effective pixel size of 130 nm after 2 × 2 binning.

### Confocal imaging

Confocal imaging was performed at the Imaging Facility of the Max Planck Institute of Biochemistry, Martinsried, on a ZEISS (Jena, Germany) LSM780 confocal laser scanning microscope equipped with a ZEISS Plan-APO 63x/NA1.46 oil immersion objective.

### DNA-PAINT super-resolution image acquisition

Cells were screened for focal adhesion formation with 488 nm laser excitation at 0.01 kW/cm^2^. After acquisition of the 488 channel, the excitation was switched to 561 nm, focal plane and TIRF angle were readjusted and imaging was subsequently performed using ~0.6–0.7 kW/cm^2^ 561 nm laser excitation. Imager strand (for sequences see Supplementary Table 1) concentration for extended sensor measurements was adjusted to 200 pM X63* imager and acquired for 50,000 Frames at 200 ms exposure time. For exchange experiments and for measuring the surface density of ligand, the channel was washed, and a P3* imager strand was introduced at 75 pM concentration. Images were acquired for 250,000 Frames at an exposure time of 100 ms using a power density of ~1.2 kW/cm^2^. For exchange experiments with actin imaging, after washing away X63* imager strand, 2 nM of Cy3B-labeled Lifeact peptide (Peptide Facility, Max-Planck Institute of Biochemistry) was introduced and imaged with ~2.1 kW/cm^2^ 561 nm laser excitation (See Supplementary Table 2 for detailed imaging conditions). Imaging was performed in 1× PCA (Sigma-Aldrich, Cat. No. 37580-25G-F), 1× PCD (Sigma-Aldrich, Cat. No. P8279-25UN), 1× Trolox (Sigma-Aldrich, Cat. No. 238813-1 G) in Buffer C (1× PBS + 500 mM NaCl). 3D imaging was performed using an astigmatism lens in the detection path as previously described^50^. Raw microscopy data was acquired using µManager^51^ (Version 2.0-gamma).

### Data analysis

DNA-PAINT analysis with Picasso 0.3.0 was mostly performed as previously reported^30^. In brief, acquired raw localization images were further processed for spot detection and fitting with the Picasso localize module. Further drift correction and alignment of exchange datasets was performed via 90 nm gold nanoparticles as fiducial markers with the Picasso render module. Focal adhesion areas were manually selected with the rectangular pick tool. Further analysis on the selected localizations and image export was performed with Picasso render and SMAP. Statistical analysis (two-sided *t*-test) was performed with Origin 2019b.

### Cluster analysis

Cluster analysis was performed with a modified Ripley’s K function by calculating the number of neighboring localizations within a 10 nm radius for each localization. Then, a gradient ascend was used to identify the cluster center exhibiting the highest number of neighbors within the 10 nm radius. All localizations within a 10 nm radius of the cluster center were assigned to this site. Detected clusters were then filtered for repetitive visits via a mean-frame analysis of occurring localizations within 20–80% of the total acquisition time as described earlier^52^. Cluster centers were then used for subsequent nearest neighbor calculations.

### Simulations

Simulations were performed with custom-written python scripts and the Picasso simulate module^30^. In brief, the experimentally observed molecular density was used to create a random distribution of positions. These positions were then loaded into Picasso simulated and raw fluorescence data was simulated using experimentally determined conditions to yield the same resolution and events per site as in experiments. For the simulations, we used a power density setting of 0.5 kW/cm^2^, otherwise the imager concentration and frame numbers were used from the respective dataset (see Supplementary Table 2). Further processing was performed with the same analysis as the experimentally acquired datasets, starting from initial spot detection and spot fitting of the simulated data as the first step.

### Axial localization analysis of hairpin vs. actin signals

For colocalization analysis between the extended hairpin and the actin filaments, areas were manually picked with the rectangular pick tool in Picasso render. Localizations were rotated using a custom script, to align the actin filament and the associated hairpin signal along the horizontal axis and the mean position in x and z of the localizations was determined in a 75 nm sliding window along the x-axis for each dataset.

### DNA hairpin probe sequence

Biotin-5′-TTTTATACATCTAGTTTTTGCGATTTTACACCGCTTTTTTGCGGTGTAAAATCGCTTTCTTCATTATT-3′-DBCO

### Unzipping force of double stranded DNA

The unzipping force at which 50% (F_1/2_) of the DNA hairpin force sensors are extended was calculated as previously described^24^. In brief, the force can be described with following formulae:

1 $${F}_{1/2}=\frac{\triangle {G}_{{fold}}+\triangle {G}_{{stretch}}}{\triangle x}$$

The free energy $\triangle {G}_{{fold}}$ was calculated using NUPACK and $\triangle {G}_{{stretch}}$ was determined as follows:

2 $${\triangle} {G}_{{stretch}}=\frac{k_{B} T L_{0}}{L_{p} 4\left(\right.1-(\frac{x}{L_{0}})}\left[3 \left( \frac{x}{L_{0}}\right)^{2}-2 \left(\frac{x}{L_{0}}\right)^{3}\right]$$

where ${k}_{B}$ is the Boltzmann constant, *T* the temperature (37 °C in our case) and *L*_*p*_ the persistence length of ssDNA (1.3 nm in our case). *L*_*0*_ is the contour length of the DNA strand and *x* the extension of the DNA hairpin from its folded state, calculated by:

3 $$x=0.44\left(n-1\right)$$

with *n* being the number of base pairs. To obtain F_1/2_, $\triangle x$, which is the displacement of the DNA hairpin during unfolding, was calculated as:

4 $$\Delta x=\left(0.44\left(n-1\right)\right)-2$$

Using these formulae, the unzipping force of our DNA hairpin sensor was estimated to be 9 pN.

### Statistics and reproducibility

All experiments were performed independently at least three times. Typical micrographs were derived from *n* = 3 independent experiments for surface hairpins shown in Fig. 2a, Supplementary Fig. 2 and 7. Cellular micrographs in Fig. 2f and h, Fig. 3a, b and f, Supplementary Fig. 8, 9a and b, 10a and d, 11 and 12a were derived from *n* = 17 independent experiments. Micrograph in Supplementary Fig. 6a and b was derived from *n* = 4 independent experiments. Micrograph for Supplementary Fig. 5 was derived from 5 simulations. Micrographs in Fig. 4a and b, Supplementary Figs. 13a, b, c, 14 and 15a, b were derived from *n* = 6 independent experiments.

### Reporting summary

Further information on research design is available in the Nature Research Reporting Summary linked to this article.

## Supplementary information

Supplementary Information

Reporting Summary

## Data Availability

Data supporting the findings of this manuscript are available from the corresponding author upon reasonable request. A reporting summary for this Article is available as a Supplementary Information file.

## Code availability

The Picasso software can be found at https://github.com/jungmannlab/picasso.
