# Supplementary material for: Super-resolved visualization of single DNA-based tension sensors in cell adhesion
Source: Nat Commun. 2021 May 4;12:2510. doi: 10.1038/s41467-021-22606-1 (PMC8097079; doi:10.1038/s41467-021-22606-1)
Supplement: Supplementary file 1 — Supplementary Information [file 41467_2021_22606_MOESM1_ESM.pdf]

# Supplementary Information

## Super-resolved visualization of single DNA-based tension sensors in cell adhesion

Thomas Schlichthaerle[1,2,3], Caroline Lindner[1,2,3], Ralf Jungmann[1,2]

[1]Faculty of Physics and Center for Nanoscience, Ludwig Maximilian University, Munich, Germany. [2]Max Planck Institute of Biochemistry, Martinsried, Germany. [3]These authors contributed equally. Correspondence should be addressed to R.J. (jungmann@biochem.mpg.de).


| | |
|---|---|
| **Supplementary Figure 1** | NUPACK secondary structure prediction of hairpin sensor probe at 37 °C |
| **Supplementary Figure 2** | 100 single closed hairpin sensors |
| **Supplementary Figure 3** | Binding events analysis of individual sites |
| **Supplementary Figure 4** | Sensor detection on the surface |
| **Supplementary Figure 5** | Simulation of randomly distributed single sites on a surface |
| **Supplementary Figure 6** | Confocal imaging of cell surface attachment |
| **Supplementary Figure 7** | Imaging of permanently accessible and opened sites of sensors underneath a cell |
| **Supplementary Figure 8** | 100 single opened hairpin sensors |
| **Supplementary Figure 9** | DNA hairpin sensor imaging underneath a cell |
| **Supplementary Figure 10** | Four additional cells showing opened hairpins below the cell |
| **Supplementary Figure 11** | Overview images of opened hairpin sensors in clusters |
| **Supplementary Figure 12** | Analysis of inner clusters |
| **Supplementary Figure 13** | Correlation of hairpin and actin signals using 3D-DNA-PAINT |
| **Supplementary Figure 14** | Actin network colocalization with opened sensor |
| **Supplementary Figure 15** | Actin network colocalization with inner clusters |
| **Supplementary Table 1** | DNA-PAINT imager strand sequences |
| **Supplementary Table 2** | Imaging Parameters |
| **Supplementary References** | |

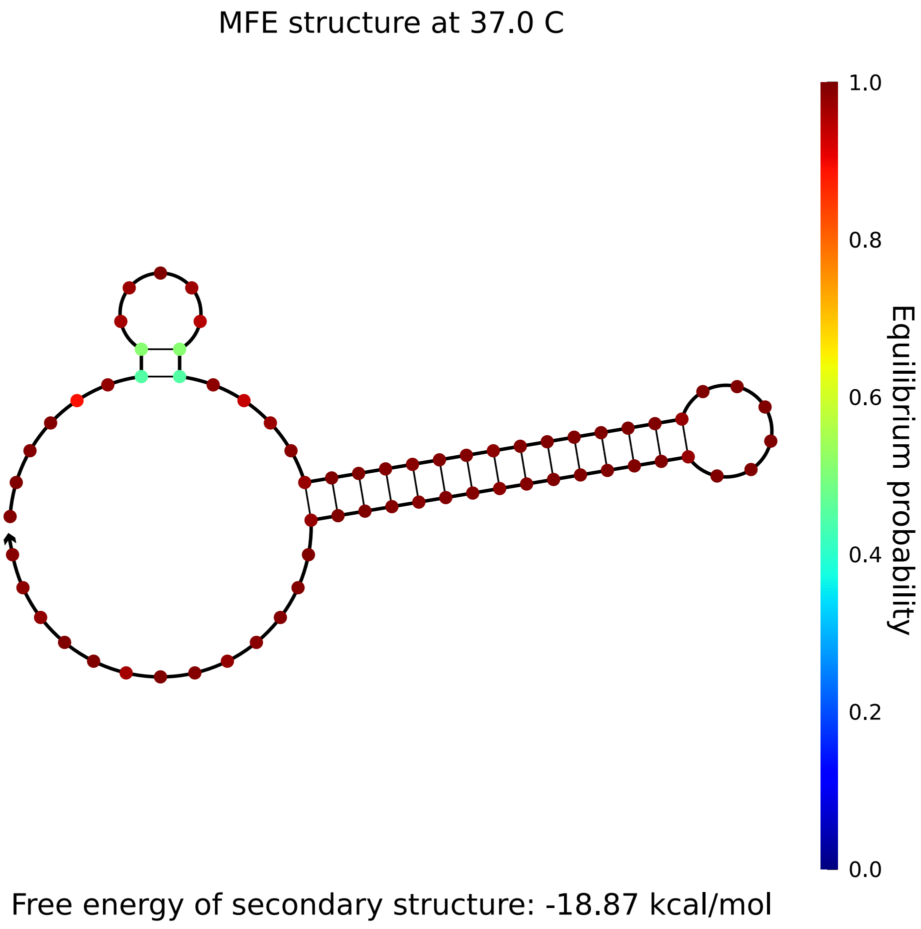


**Supplementary Figure 1 | NUPACK[1] secondary structure prediction of hairpin sensor probe at 37 °C.**

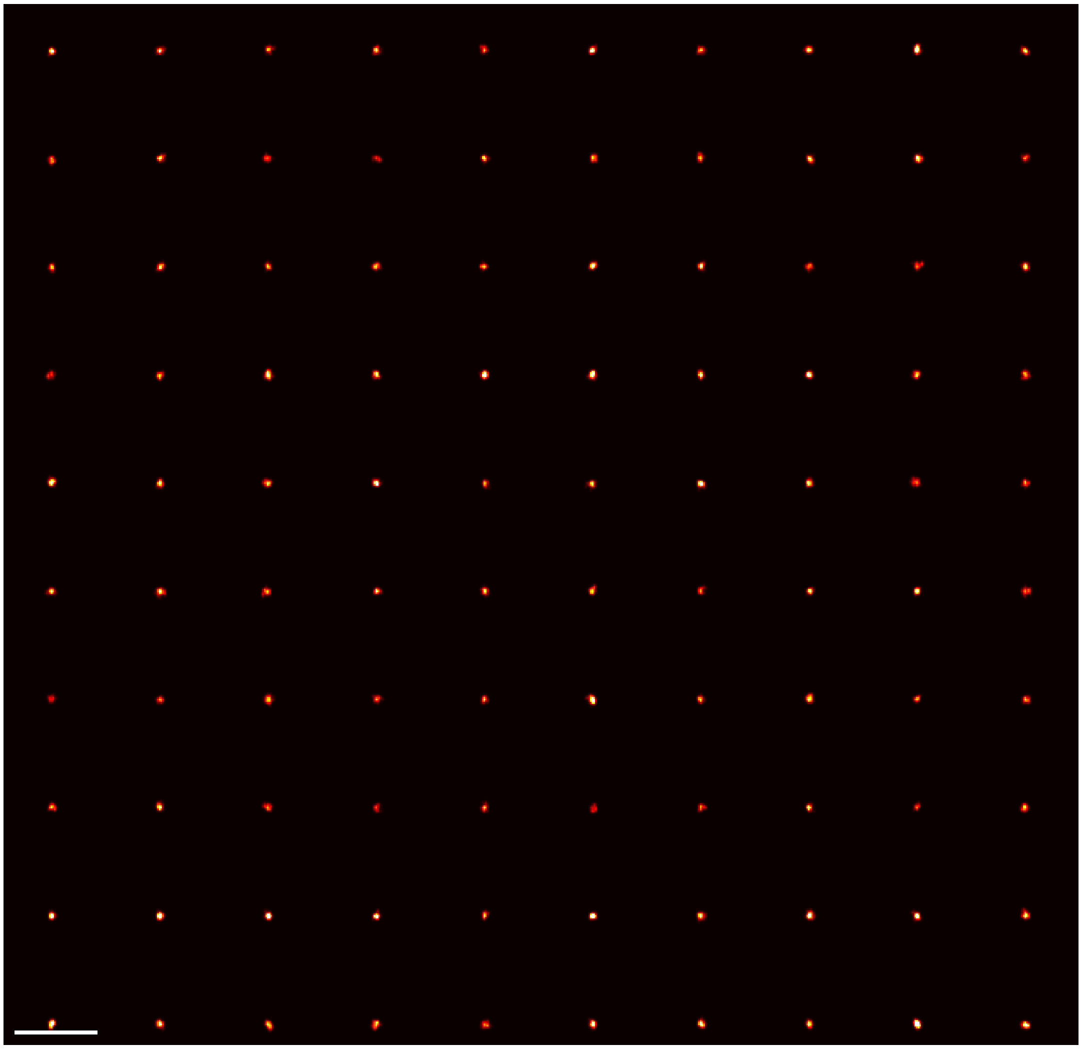

**Supplementary Figure 2 | 100 single closed hairpin sensors.** Scale bar: 200 nm

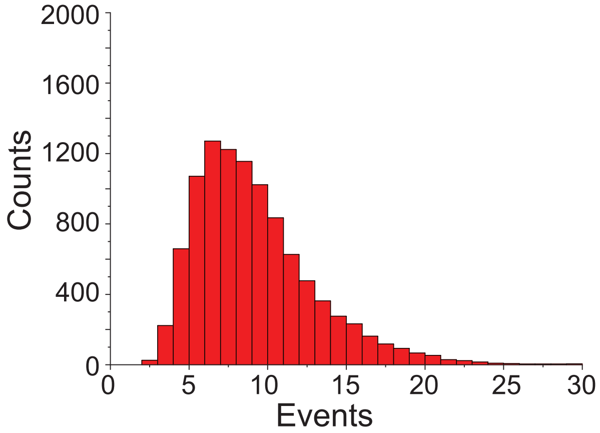


**Supplementary Figure 3 | Binding events analysis of individual sites.** Analysis of binding events of the permanently accessible part of the hairpin sensor of single sites on the surface shows a unimodal distribution.

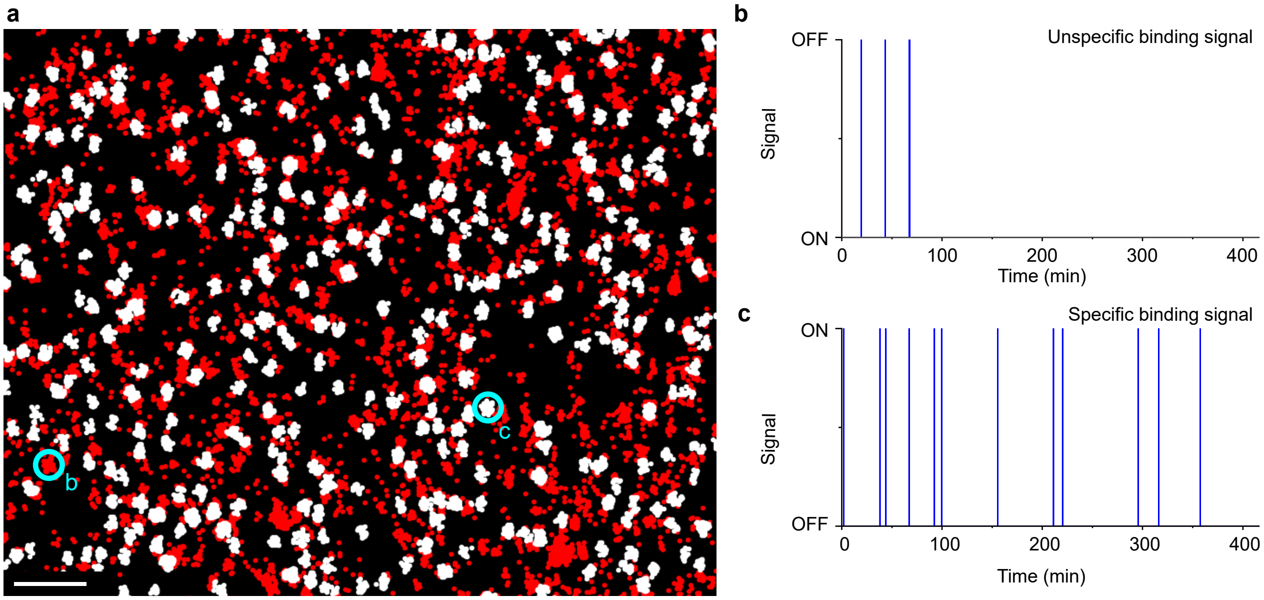


**Supplementary Figure 4 | Sensor detection on the surface.** (**a**) Overlay of detected sites from the clustering algorithm with the raw localization data. (**b**) Signal of a filtered-out site shows non-repetitive visits over the whole image acquisition, which most likely stems from non-specific interactions of imager strands with the surface. The algorithm successfully removed these unwanted signals. (**c**) Signal of a detected site shows repetitive binding events over the whole image acquisition and is thus kept a true signal. Scale bar: 100 nm

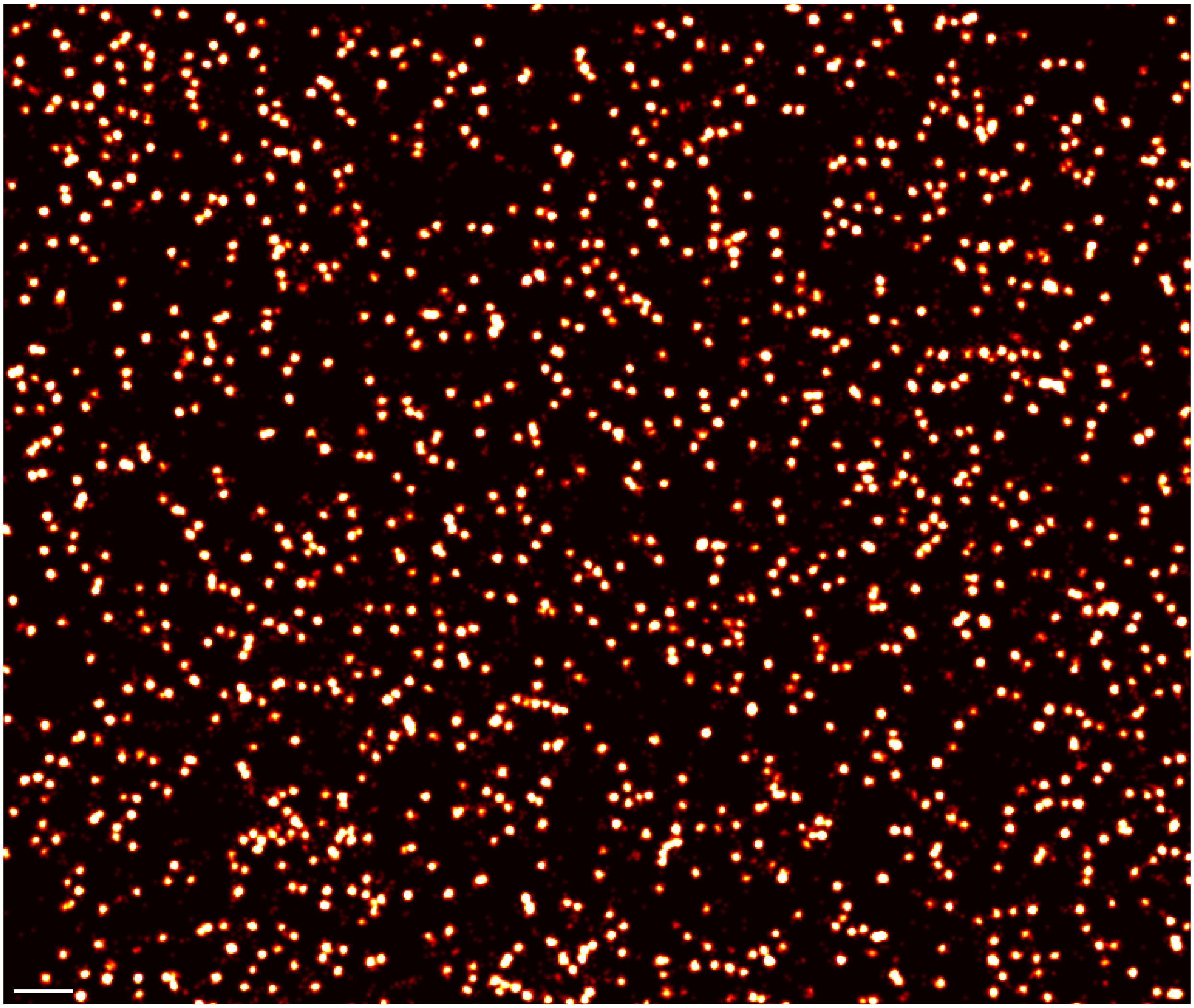

**Supplementary Figure 5 | Simulation of randomly distributed single sites on a surface.** A molecular density of 422 particles per μm$^2$ was used. Scale bar: 100 nm

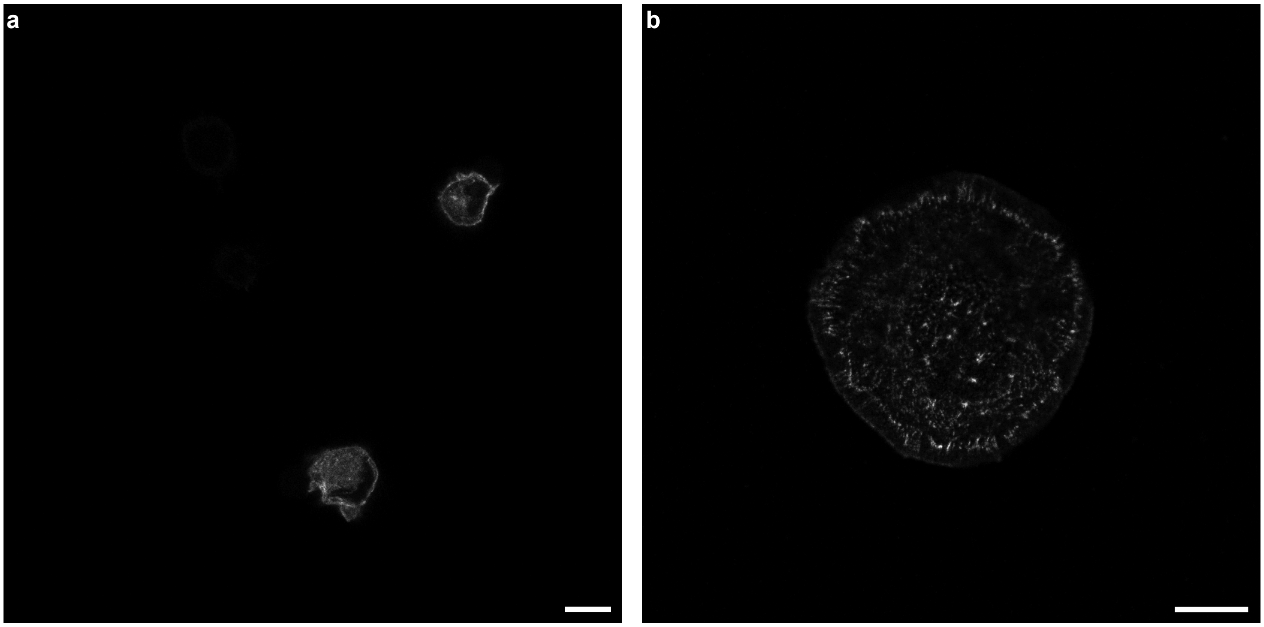


**Supplementary Figure 6 | Confocal imaging of cell surface attachment.** (**a**) Cells did not spread or form adhesions on PEG750 surfaces without any sensor on the surface. (**b**) Cells spreaded and formed adhesions as indicated by a distinct talin signal after 25 min on PEG750 surfaces with biotin-anchored cRGD DNA hairpin sensors. Scale bars: 10 µm

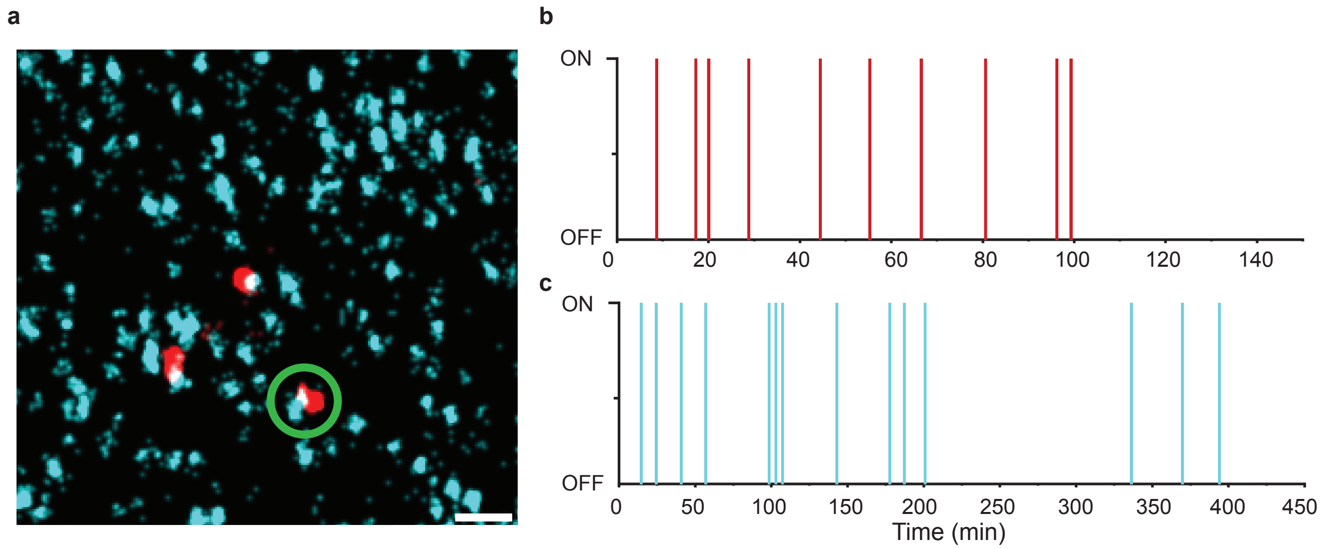


**Supplementary Figure 7 | Colocalization of opened hairpin (red) with permanently accessible site of hairpin sensors (blue) below a cell. (a)** Exchange-PAINT imaging of unfolded (red) and permanently accessible (cyan) hairpin site below a cell (same data as in Figure 2). (**b**) Repetitive imaging trace of unfolded sensor site shows specific signal (red localizations from green circle in a). (**c**) Repetitive imaging trace of permanently accessible part of sensor site shows specific signal (cyan localizations from green circle in a). Scale bar: 50 nm

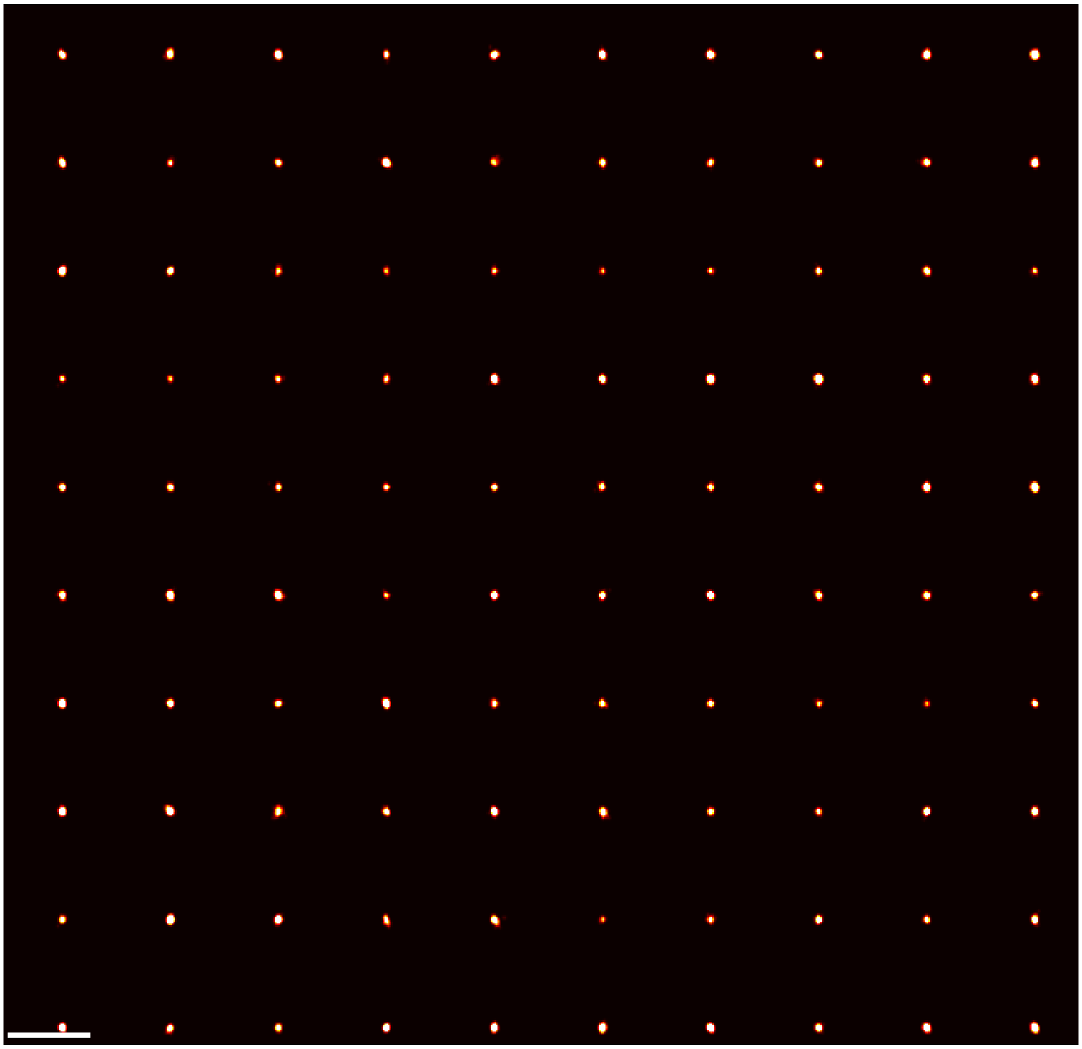

**Supplementary Figure 8 | 100 single opened hairpin sensors.** Scale bar: 200 nm

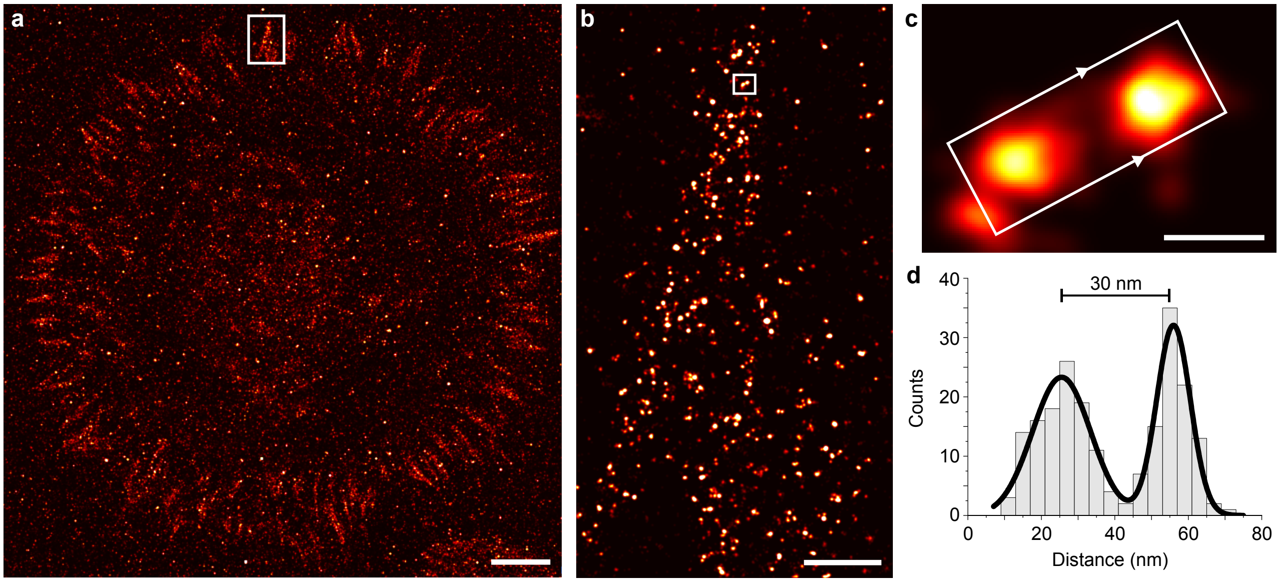


**Supplementary Figure 9 | DNA hairpin sensor imaging underneath a cell.** (**a**) Overview of mechanically unfolded hairpins underneath a cell shows enriched signals in focal adhesions. (**b**) Zoom-in of focal adhesion area highlighted in **a**. (**c**) Zoom-in of highlighted area in **b** shows two closely spaced sensors. (**d**) Cross-sectional histogram analysis yields a distance of 30 nm. Scale bars: 5 µm (**a**), 500 nm (**b**), 20 nm (**c**).

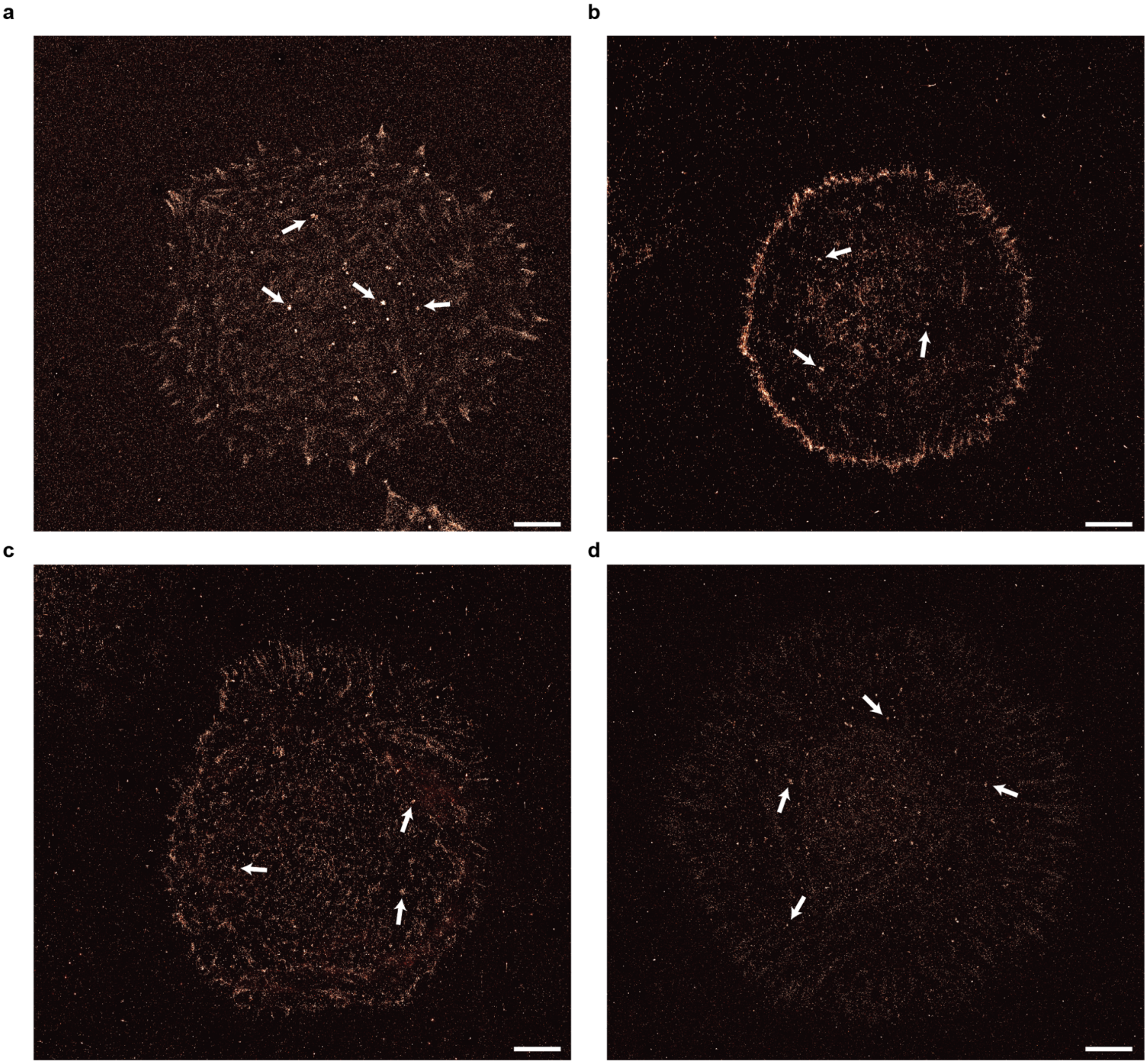


**Supplementary Figure 10 | Four additional cells showing mechanically opened hairpins below the cell.** Four additional cells, showing opened hairpins below the cell. Scale bars: 5 µm

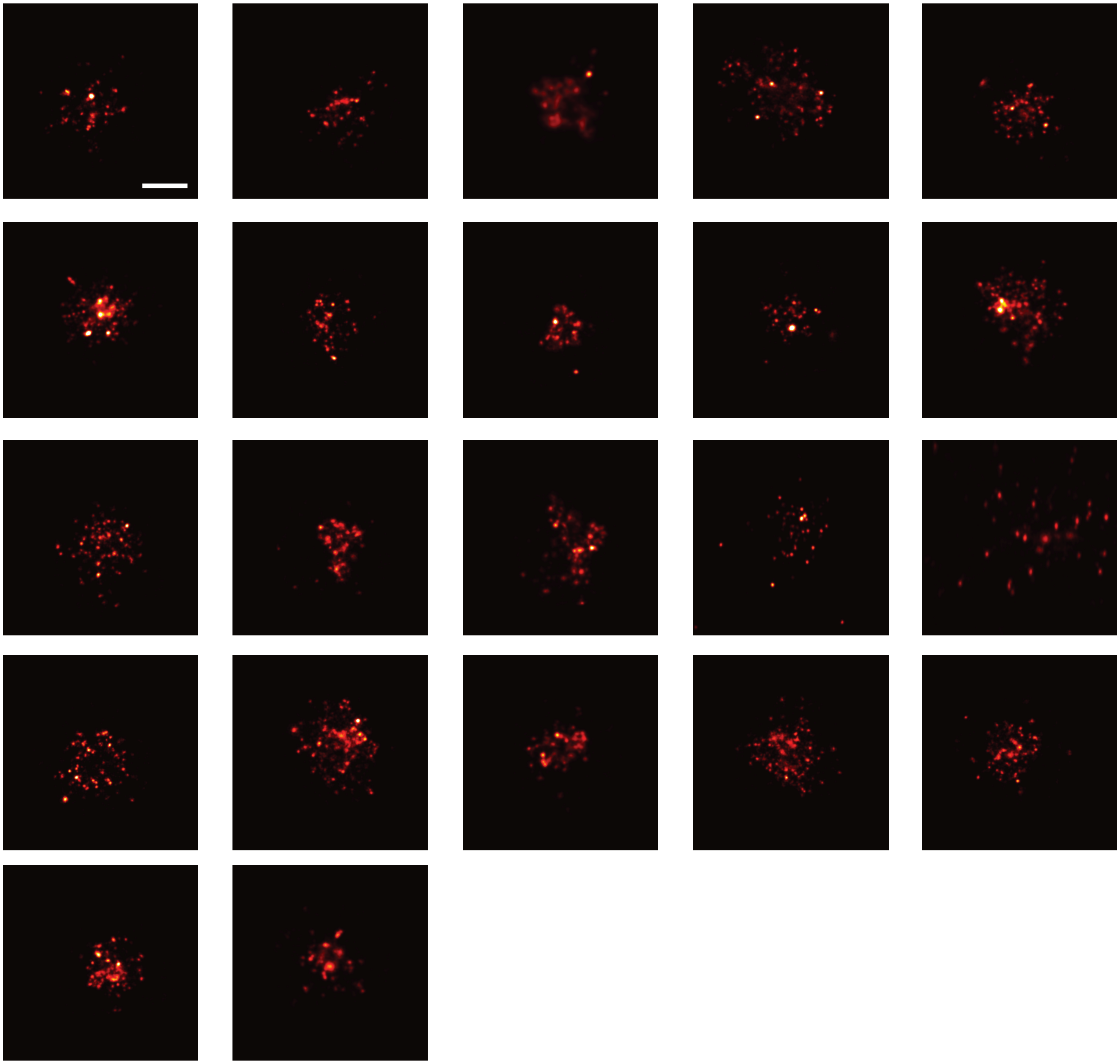

**Supplementary Figure 11 | Overview images of opened hairpin sensors in clusters**. All clusters show repetitive binding traces. Scale bar: 200 nm

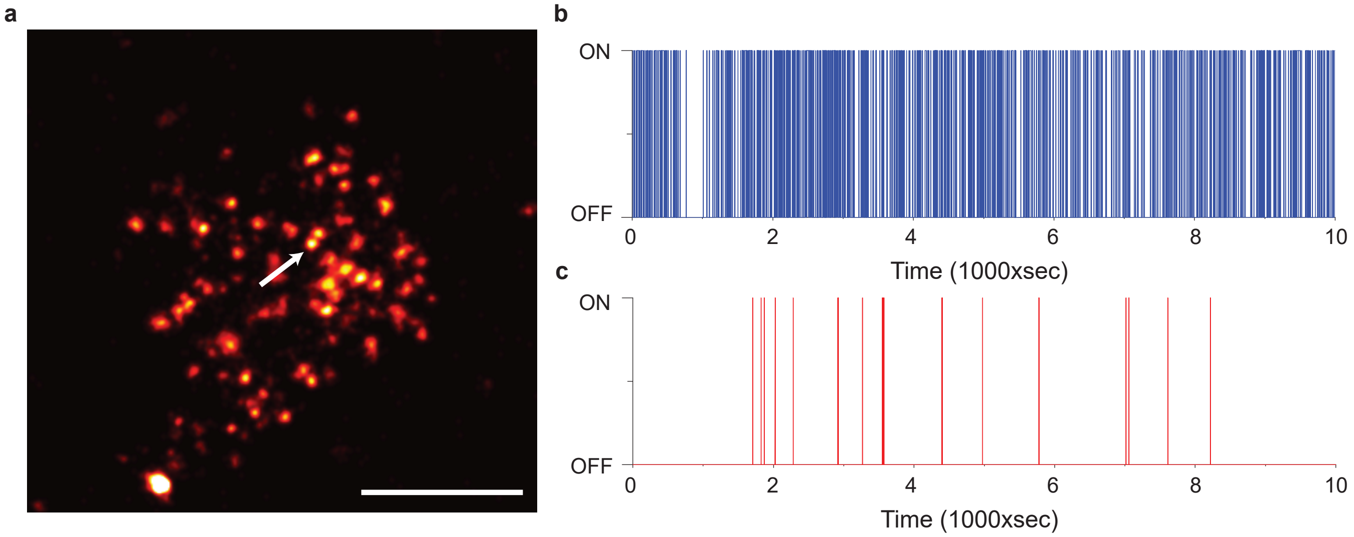


**Supplementary Figure 12 | Analysis of inner clusters.** (**a**) Exemplary "inner" cluster of opened hairpins. (**b**) Visits of DNA imager strands over the whole time of image acquisition at the whole cluster shows repetitive binding. (**c**) Analyzing the repetitive visits of a single site in the cluster shows repetitive binding. Scale bar: 200 nm

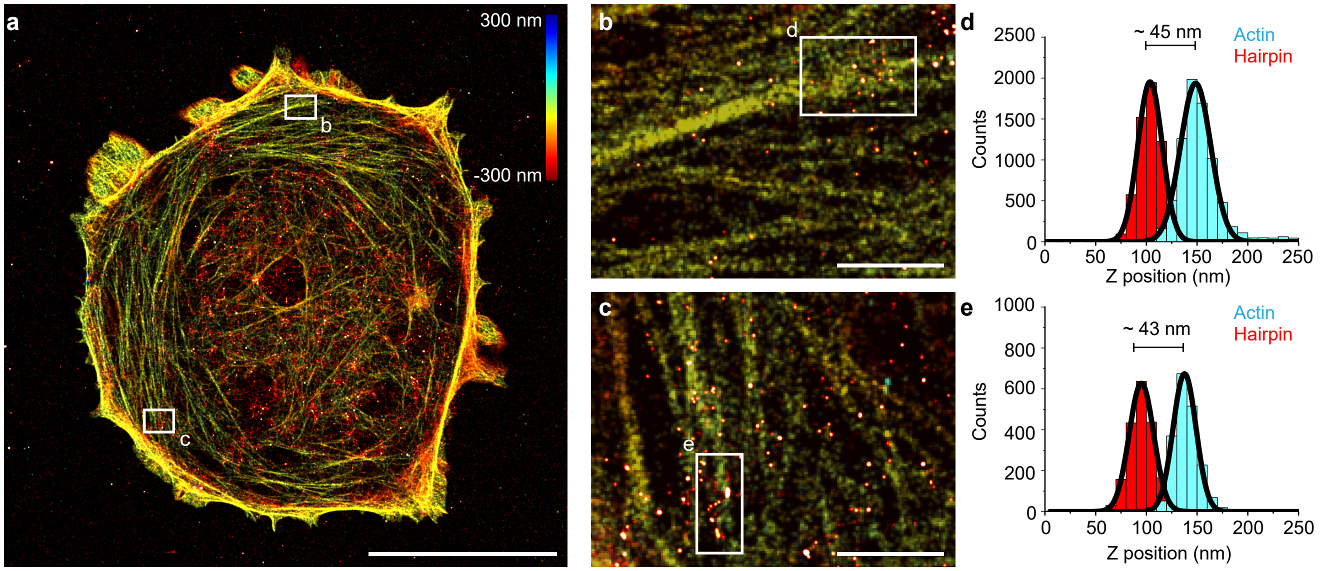


**Supplementary Figure 13 | Correlation of hairpin and actin signals using 3D-DNA-PAINT.** (**a**) Overview of actin architecture in comparison to extended hairpin signal. Colormap indicates axial position ranging from -300 nm to 300 nm for actin. (**b**) Zoom-in of upper area highlighted in **a** reveals opened hairpins beneath actin filaments. (**c**) Zoom-in of lower area highlighted in **a** reveals opened hairpins at the tip of actin filaments. (**d**) Axial localization histogram analysis of area highlighted in **b** reveals a distance of ~45 nm between opened hairpin sensor and actin filaments. (**e**) Same analysis for area highlighted in **c** shows ~43 nm between opened hairpins and actin filaments. Scale bars: 10 µm (**a**), 1 µm (**b** and **c**).

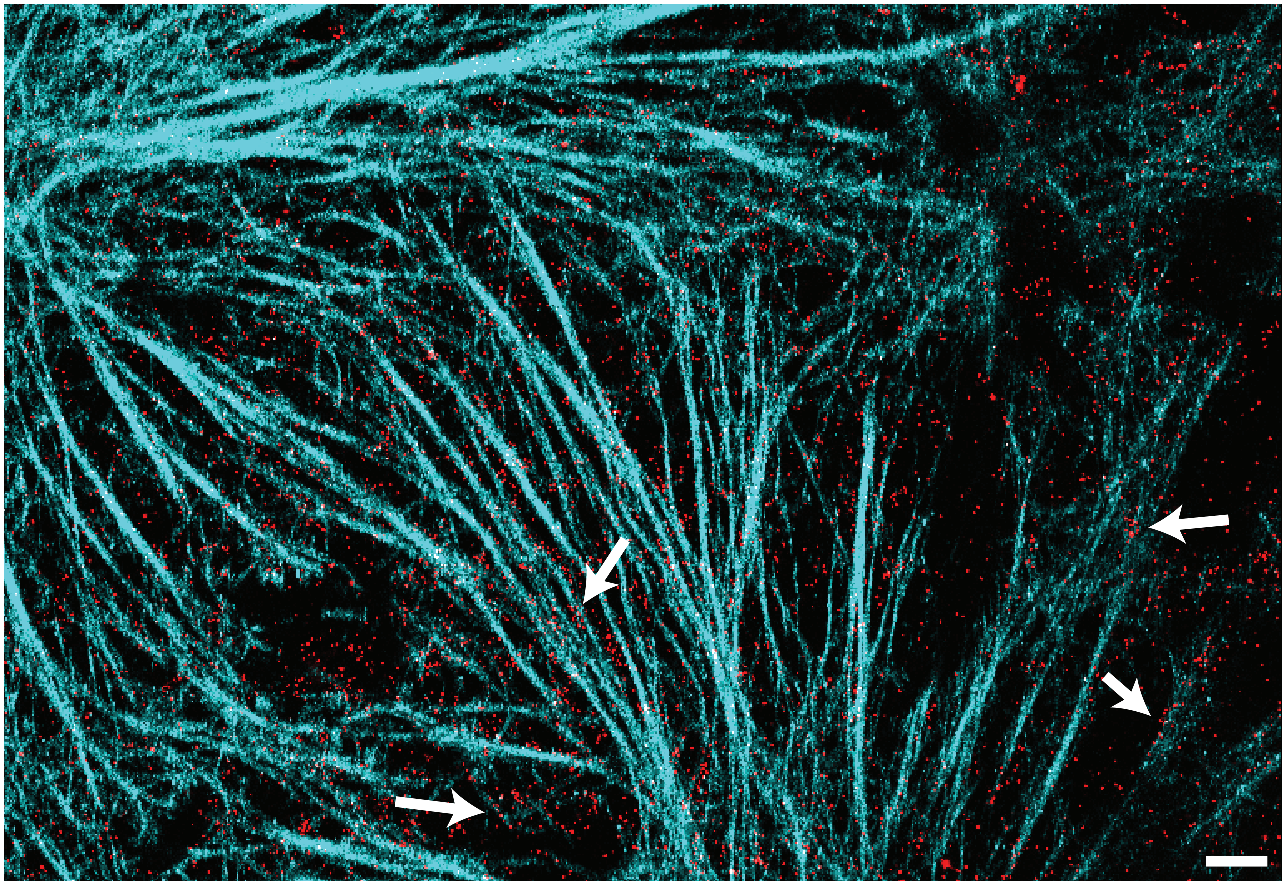

**Supplementary Figure 14 | Actin network colocalization with opened sensor.** Opened sensors (red) can be found along actin fibers (blue) as well as at fiber tips. Arrows indicate opened hairpin sensors along fibers. Scale bar: 1 µm

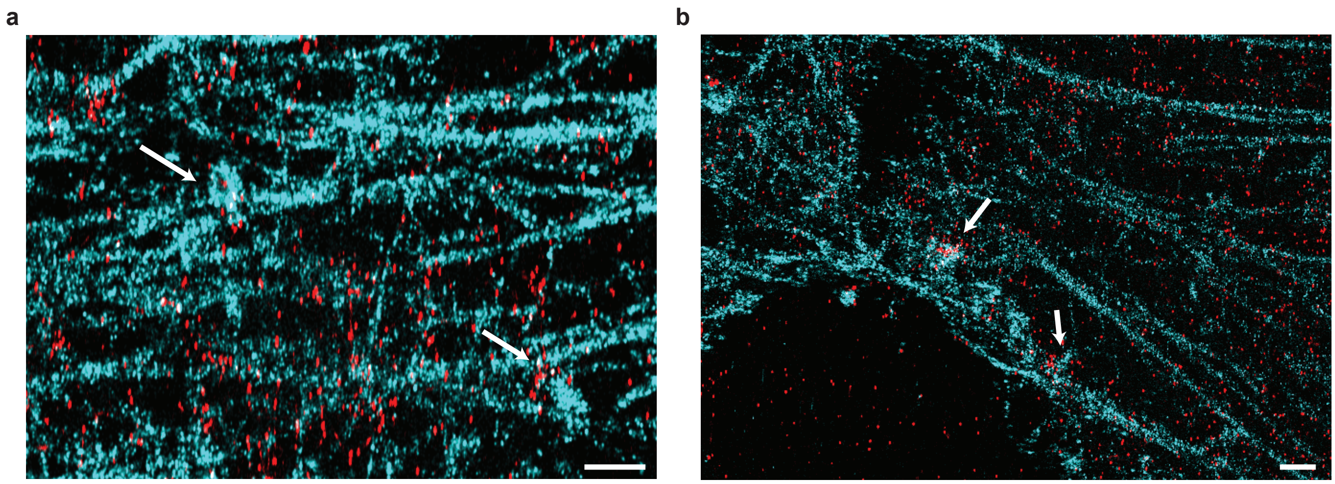


**Supplementary Figure 15 |Actin network colocalization with inner clusters.** (**a**) Opened hairpin signals (red) with actin filaments (blue), shows colocalization of actin bundles with clusters. (**b**) Opened hairpin signals (red) with actin filaments (blue) shows colocalization with actin bundles. Scale bars: 500 nm

## Supplementary Table 1 | DNA-PAINT imager strand sequences

| Imager name | Sequence | 5'-mod | 3'-mod | Vendor |
|---|---|---|---|---|
| X63* | GGTGTAAAAT | None | Cy3b | Eurofins Genomics |
| P3* | GTAATGAAGA | none | Cy3b | Eurofins Genomics |

## Supplementary Table 2 | Imaging parameters

| Dataset | Parameters | Laser power @561 nm | Localization precision (NeNA) |
|---|---|---|---|
| Figure 2a, Supp. Figure 2,4,7a,c | 100 ms, 3D, 250,000 Frames, 75 pM P3* | 1.2 kW/cm$^2$ | 4.1 nm |
| Figure 2f, Supp. Figure 7a,b, 8 | 150 ms, 3D, 60,000 Frames, 200 pM, X63* 9 nt | 0.6 kW/cm$^2$ | 4.4 nm |
| Figure 3, Supp. Figure 11, 12 | 200 ms, 2D, 50,000 Frames, 200 pM, X63* 9 nt | 0.7 kW/cm$^2$ | 4.3 nm |
| Figure 4 , Supp. Figure 14 | Lifeact: 50 ms, 3D, 300,000 Frames, 2 nM, Lifeact-Cy3b<br>Hairpin: 200 ms, 3D, 50,000 Frames, 200 pM, X63* 9 nt | 2.1 kW/cm$^2$<br>0.7 kW/cm$^2$ | 5.6 nm<br>6.2 nm |
| Supp. Figure 9, Supp. Figure 9d | 200 ms, 2D, 50,000 Frames, 150 pM, X63* 9 nt | 0.7 kW/cm$^2$ | 4.8 nm |
| Supp. Figure 10a | 200 ms, 2D, 50,000 Frames, 200 pM, X63* 9nt | 1.4 kW/cm$^2$ | 4.8 nm |
| Supp. Figure 10b | 150 ms, 3D, 60,000 Frames, 200 pM, X63* 9nt | 0.7 kW/cm$^2$ | 7.5 nm |
| Supp. Figure 10c | 150 ms, 3D, 60,000 Frames, 200 pM, X63* 9nt | 0.6 kW/cm$^2$ | 5.6 nm |
| Supp. Figure 13 | Lifeact: 50 ms, 3D, 300,000 Frames, 2.5 nM, Lifeact-Cy3b<br>Hairpin: 100 ms, 3D, 80,000 Frames, 200 pM, X63* 9nt | 2.1 kW/cm$^2$<br>0.7 kW/cm$^2$ | 4.3 nm<br>8.5 nm |
| Supp. Figure 15a | Lifeact: 50 ms, 3D, 300,000 Frames, 2.5 nM, Lifeact-Cy3b<br>Hairpin: 150 ms, 3D, 60,000 Frames, 200 pM, X63* 9nt | 2.1 kW/cm$^2$<br>0.6 kW/cm$^2$ | 6.3 nm<br>7.5 nm |
| Supp. Figure 15b | Lifeact: 50 ms, 3D, 300,000 Frames, 2.5 nM, Lifeact-Cy3b<br>Hairpin: 150 ms, 3D, 60,000 Frames, 200 pM, X63* 9nt | 2.1 kW/cm$^2$<br>0.6 kW/cm$^2$ | 5.4 nm<br>6.6 nm |

**Supplementary References**

1. Zadeh, J.N. et al. NUPACK: Analysis and design of nucleic acid systems. *J Comput Chem* **32**, 170-173 (2011).
