# Supplementary material for: Super-resolved visualization of single DNA-based tension sensors in cell adhesion
Source: Nat Commun. 2021 May 4;12:2510. doi: 10.1038/s41467-021-22606-1 (PMC8097079; doi:10.1038/s41467-021-22606-1)
Supplement: Supplementary file 2 — Reporting Summary [file 41467_2021_22606_MOESM2_ESM.pdf]

nature research

Corresponding author(s): Ralf Jungmann
Last updated by author(s): Mar 9, 2021

Reporting Summary

Nature Research wishes to improve the reproducibility of the work that we publish. This form provides structure for consistency and transparency in reporting. For further information on Nature Research policies, see our Editorial Policies and the Editorial Policy Checklist.

Statistics

For all statistical analyses, confirm that the following items are present in the figure legend, table legend, main text, or Methods section.

n/a	Confirmed	
☐	☒	The exact sample size (n) for each experimental group/condition, given as a discrete number and unit of measurement
☐	☒	A statement on whether measurements were taken from distinct samples or whether the same sample was measured repeatedly
☒	☐	The statistical test(s) used AND whether they are one- or two-sided<br>*Only common tests should be described solely by name; describe more complex techniques in the Methods section.*
☒	☐	A description of all covariates tested
☒	☐	A description of any assumptions or corrections, such as tests of normality and adjustment for multiple comparisons
☐	☒	A full description of the statistical parameters including central tendency (e.g. means) or other basic estimates (e.g. regression coefficient) AND variation (e.g. standard deviation) or associated estimates of uncertainty (e.g. confidence intervals)
☒	☐	For null hypothesis testing, the test statistic (e.g. F, t, r) with confidence intervals, effect sizes, degrees of freedom and P value noted<br>*Give P values as exact values whenever suitable.*
☒	☐	For Bayesian analysis, information on the choice of priors and Markov chain Monte Carlo settings
☒	☐	For hierarchical and complex designs, identification of the appropriate level for tests and full reporting of outcomes
☒	☐	Estimates of effect sizes (e.g. Cohen's d, Pearson's r), indicating how they were calculated

Our web collection on statistics for biologists contains articles on many of the points above.

Software and code

Policy information about availability of computer code

Data collection	Raw microscopy data was acquired using µManager (Version 2.0-gamma) (Edelstein, A., Amodaj, N., Hoover, K., Vale, R. & Stuurman, N. Curr. Protoc. Mol. Biol. 14.20 (2010))
Data analysis	Custom analysis software Picasso was used to process raw super-resolution data. The software is open source and was described in detail in the following publication: Nature Protocols volume 12, pages 1198–1228 (2017) doi:10.1038/nprot.2017.024<br>Origin Pro 2019b was used for data fitting and plotting.

For manuscripts utilizing custom algorithms or software that are central to the research but not yet described in published literature, software must be made available to editors and reviewers. We strongly encourage code deposition in a community repository (e.g. GitHub). See the Nature Research guidelines for submitting code & software for further information.

Data

Policy information about availability of data

All manuscripts must include a data availability statement. This statement should provide the following information, where applicable:
- Accession codes, unique identifiers, or web links for publicly available datasets
- A list of figures that have associated raw data
- A description of any restrictions on data availability

All raw data are available upon request from the authors.

Field-specific reporting

Please select the one below that is the best fit for your research. If you are not sure, read the appropriate sections before making your selection.

- [x] Life sciences
- [ ] Behavioural & social sciences
- [ ] Ecological, evolutionary & environmental sciences

For a reference copy of the document with all sections, see nature.com/documents/nr-reporting-summary-flat.pdf

Life sciences study design

All studies must disclose on these points even when the disclosure is negative.

Sample size	Proof of concept for working hairpin was determined with >10 independent repeat experiments (not all data is shown) with high success rate. Statistical analysis for engaged hairpin versus simulation was taken from n=4 experiments, with each containing more then 1000 individual datapoints.
Data exclusions	No data were excluded.
Replication	All replications were successful. All experiments were performed independently at least three times.
Randomization	No grouping of experiments or samples were performed
Blinding	Blinding is not applicable to the study since no grouping was performed

Reporting for specific materials, systems and methods

We require information from authors about some types of materials, experimental systems and methods used in many studies. Here, indicate whether each material, system or method listed is relevant to your study. If you are not sure if a list item applies to your research, read the appropriate section before selecting a response.

Materials & experimental systems

n/a	Involved in the study	
x		Antibodies
	x	Eukaryotic cell lines
x		Palaeontology and archaeology
x		Animals and other organisms
x		Human research participants
x		Clinical data
x		Dual use research of concern

Methods

n/a	Involved in the study	
x		ChIP-seq
x		Flow cytometry
x		MRI-based neuroimaging

Eukaryotic cell lines

Policy information about cell lines

Cell line source(s)	Double knockout fibroblasts deficient for talin-1 and talin-2 (Tln1-/- Tln2-/-) reconstituted via retroviral infection with hTalin1-YPet[447] as described in Austen et. al. "Extracelullar rigidity sensing by talin isoform-specific mechanical lingakes" Nat Cell Biol 17, 1597-1606 (2015). Cell line kindly provided by Prof. Dr. Carsten Grashoff
Authentication	The cell lines were not authenticated.
Mycoplasma contamination	All cell lines have been tested negative for mycoplasma contamination.
Commonly misidentified lines (See ICLAC register)	No commonly misidentified cell lines were used.
